# Genetic differentiation and recombination among geographic populations of the fungal pathogen *Colletotrichum truncatum* from chili peppers in China

**DOI:** 10.1111/eva.12233

**Published:** 2014-12-13

**Authors:** Yongzhao Diao, Can Zhang, Jianping Xu, Dong Lin, Li Liu, Olivo G Mtung'e, Xili Liu

**Affiliations:** 1 Department of Plant Pathology, College of Agriculture and Biotechnology, China Agricultural UniversityBeijing, China; 2 Department of Biology, McMaster UniversityHamilton, ON, Canada

**Keywords:** clustering analyses, *Colletotrichum truncatum*, genetic differentiation, phylogeny, population structure, private alleles

## Abstract

*Colletotrichum truncatum* is an extremely important fungal pathogen. It can cause diseases both in humans and in over 460 plant species. However, little is known about its genetic diversity within and among populations. One of the major plant hosts of *C. truncatum* is pepper, and China is one of the main pepper-producing countries in the world. Here, we propose the hypotheses that geography has a major influence on the relationships among populations of *C. truncatum* in China and that infections in different populations need to be managed differently. To test these hypotheses, we obtained and analyzed 266 *C. truncatum* isolates from 13 regions representing the main pepper-growing areas throughout China. The analysis based on nine microsatellite markers identified high intrapopulation genetic diversity, evidence of sexual recombination, and geographic differentiation. The genetic differentiation was positively correlated with geographic distance, with the southern and northern China populations grouped in two distinct clusters. Interestingly, isolates collected from the pepper-breeding center harbored the most private alleles. The results suggest that the geographic populations of *C. truncatum* on peppers in China are genetically differentiated and should be managed accordingly. Our study also provides a solid foundation from which to further explore the global genetic epidemiology of *C. truncatum* in both plants and humans.

## Introduction

Species in the Ascomycete fungal genus *Colletotrichum* are common in the environment and important plant pathogens. Many species in this genus can cause not only anthracnose and blights on the aerial parts of growing crop plants but also postharvest rots (Bailey and Jeger 1992; Dean et al. 2012). Anthracnose is an important disease of chili peppers and other peppers (Vos and Frinking 1997; Harp et al. 2008; Than et al. 2008; Montri et al. 2009). China is a major producer of fresh and dried chili, and anthracnose causes yield losses of up to 30–40% on chili and other peppers in the country (http://faostat.fao.org) (Qing et al. 2005).

A major species in genus *Colletotrichum* causing anthracnose of peppers is *C. truncatum* (syn. *C. capsici*) (Damm et al. 2009). Aside from infecting peppers, *C. truncatum* has been reported to infect more than 460 plant species (http://nt.ars-grin.gov/fungaldatabases/) (Sutton et al. 1992; Shenoy et al. 2007; Damm et al. 2009; Yang et al. 2009; Wikee et al. 2011; Huang et al. 2013; Diao et al. 2014). In addition, *C. truncatum* can also cause mycotic keratitis and endophthalmitis in humans (Shivaprakash et al. 2011). *Colletotrichum truncatum* is generally seed-borne but can also be dispersed by wind and rain (Ranathunge et al. 2012). Its dominant reservoirs are soil and infected host debris and can survive at least 48 months on infected debris in soil (Ishaque and Talukdar 1967; Vos and Frinking 1997; Cannon et al. 2012; Ranathunge et al. 2012). However, despite its agricultural, ecological, and medical implications, relatively little is known about the epidemiology and population genetics of this fungus. This study examines the population structure of *C. truncatum* on chili peppers in China.

Although asexual reproduction predominates in the majority of plant-pathogenic fungi, many species undergo regular sexual cycles (Milgroom 1996). In the case of *C. truncatum*, however, the identity of the sexual stage is still unclear (Damm et al. 2009; Hyde et al. 2009) and whether sexual reproduction occurs in natural populations of *C. truncatum* remains to be determined.

Chili peppers are grown extensively in many regions in China. As a result, the populations of *C. truncatum* on chili peppers in China differ significantly in their ecological, geographic, and climatic conditions. For example, the climate for chili pepper-growing regions in China extends across tropical, subtropical, and temperature zones. In addition, there are several large mountains within its distribution and production range that could act as potential barriers for gene flow between geographic populations. Several previous studies have used ISSR (Ratanacherdchai et al. 2010; Mahmodi et al. 2013), RAPD (Browning et al. 1999; Chen et al. 2002), and microsatellite (Ranathunge et al. 2009; Rampersad 2013; Sharma et al. 2014) markers to analyze strains of *C. truncatum* and other *Colletotrichum* species. Ranathunge et al. (2009) developed 27 microsatellite markers and determined the diversity of 52 *C. truncatum* isolates from India, Sri Lanka, and Thailand. For *Colletotrichum graminicola*, random amplified polymorphic DNA (RAPD) marker analysis of *C. graminicola* isolates from turf grass revealed a high degree of genetic similarity among isolates recovered from the same host (Browning et al. 1999). However, due to limitations in sample size, experimental design, data analysis, and/or reliability and reproducibility of markers (Schlötterer 2004), inferences about the contributions of long-distance geographic separations to *C. truncatum* population genetic variation have not been determined.

The objectives of this study are to use microsatellite markers to analyze populations of *C. truncatum* from chili peppers across the main growing regions in China. We test the hypothesis that geographic populations of *C. truncatum* from chili peppers from different regions in China shall be genetically differentiated. Based on climate and geographic factors, we propose that the biggest contributing factor to the genetic and phenotypic differences may be latitude, between the southern and northern populations. In addition, we investigated whether natural populations of *C. truncatum* show evidence for recombination.

## Materials and methods

### Fungal samples

**Figure 1:**
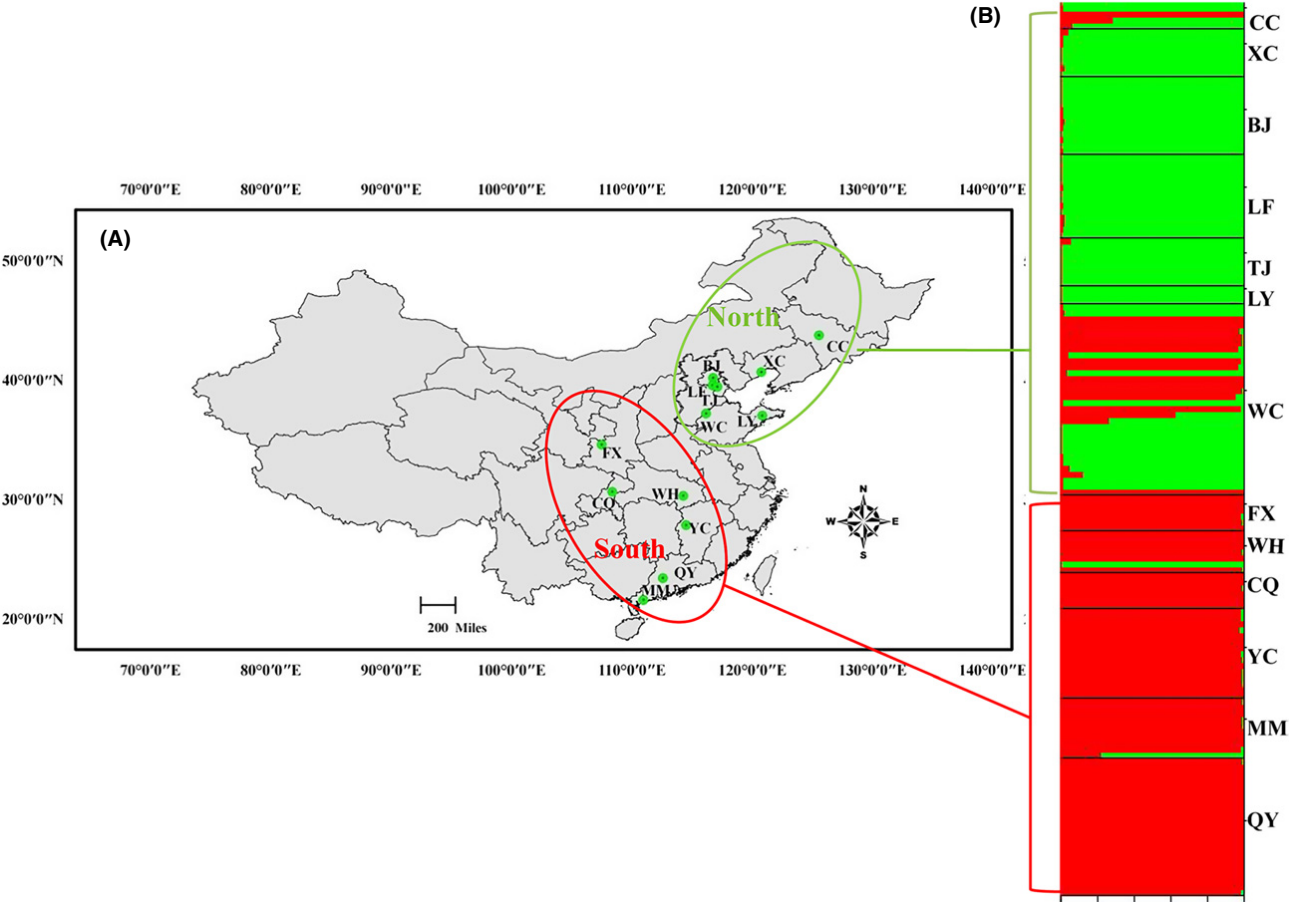
Population structure of *Colletotrichum truncatum* based on the program STRUCTURE, and sampling location of the 13 populations. (A) Map showing all sampling locations. (B) Two clusters (*K *=* *2) were identified from 13 populations based on calculations from Evanno et al. (2005), where members of the southern and northern populations formed two distinct clusters. Black line separate isolates sampled from different locations.

A total of 266 isolates of *C. truncatum* were collected from 13 locations in China (Fig.1A, Table1). Samples from each location constitute one geographic population of the pathogen. The locations were widely distributed across the country, spanning an area of about 2926 km from south to north and 1534 km from east to west and covering 11 provinces. All the isolates were collected with a hierarchical sampling method similar to that described in Kohli et al. (1995). For each geographic population, we choose five fields and sampling was performed on a diagonal transect with five locations in every field for a total of 25 chili fruits collected from each field. All isolates were obtained from pepper fruits except those from Yi Chun (the YC population) in Jiangxi province, which were from pepper leaves. We also tried to collect isolates of *C. truncatum* from other plants located close to the pepper fields, but we failed to obtain any isolates. The sample sizes and geographic coordinates for the 13 populations are shown in Table1. All isolates were obtained between 2011 and 2013 according to the method described by Cai et al. (2009).

**Table 1:** Summary information for the *Colletotrichum truncatum* populations analyzed in this study

Population code	Location (county, province)	Host tissue	Year	Number of isolates	Longitude (East)	Latitude (North)
QY	Qingyuan, Guangdong	Fruit	2013	49	23.38	112.48
MM	Maoming, Guangdong	Fruit	2013	13	21.55	110.88
YC	Yichun, Jiangxi	Leaves	2011	20	27.81	114.41
CQ	Chongqing	Fruit	2013	23	30.6	108.29
WH	Wuhan, Hubei	Fruit	2013	25	30.28	114.19
FX	Fengxiang, Shaanxi	Fruit	2011	12	34.55	107.4
WC	Wucheng, Shandong	Fruit	2011	43	37.16	116.08
LY	Laiyang, Shandong	Fruit	2011	10	36.99	120.74
TJ	Tianjin	Fruit	2012	11	39.4	117.01
LF	Langfang, Hebei	Fruit	2011	20	39.52	116.61
BJ	Beijing	Fruit	2011	19	40.15	116.65
XC	Xingcheng, Liaoning	Fruit	2012	16	40.63	120.74
CC	Changchun, Jilin	Fruit	2012	5	43.71	125.54
Total				266	21.55–43.71	107.4–125.54

### Identification of the isolates

Isolates of *C. truncatum* were identified based on morphological characteristics, and identifications were confirmed with DNA sequence data for representative isolates from each of the geographic populations. Briefly, the following six genes were sequenced for 14 isolates from the 13 populations (at least one isolate from each population): the internal transcribed spacer (ITS) region and genes encoding glyceraldehydes-3-phosphate dehydrogenase (GAPDH), a part of actin (ACT), chitin synthase 1 (CHS-1), beta-tubulin (Tub2), and histone3 (HIS3). The primers used and the PCR conditions followed the description by Damm et al. (2009). The phylogeny tree from six genes were analyzed together with the ex-epitype strain of *C. truncatum* (CBS 151.35) and other two *C. truncatum* stains (CBS136.30, CBS141.79) as well as several closely related species (Damm et al. 2009). Phylogenetic analysis was conducted using Maximum likelihood (ML) methods with MEGA5 (Tamura et al. 2011). The best evolutionary model was also determined with MEGA5. The robustness of the trees was evaluated by 1000 bootstrap replications. Sequences derived in this study were deposited in GenBank (Table S1).

### DNA extraction

Purified isolates were grown for 4 days on PDA plates before the mycelia were removed and placed in a 2-mL centrifuge tube containing a steel ball (5 mm diameter). The tubes were frozen in liquid nitrogen, and a Mixer Mill (MM400; Retsch, Haan, Germany) was used to grind the frozen mycelia to a fine powder. DNA was extracted using a 2% CTAB (cetyl trimethylammonium bromide) method (Murray and Thompson 1980) with minor modifications. Briefly, the suspension of the powder in CTAB was subjected to phenol chloroform isoamyl alcohol (v/v/v, 25:24:1) extraction and isopropanol precipitation. The extracted DNA was suspended in 50 μL of distilled water.

### Microsatellite analysis

Based on the patterns of polymorphism described previously (Ranathunge et al. 2009), nine microsatellite markers were chosen to analyze strains of *C. truncatum* for this study (Table2). The PCR conditions used for the amplification were the same as those described by Ranathunge et al. (2009), except that the annealing temperature for marker CCSSR1 was 64°C. The primers were labeled with three fluorescent dyes: FAM (CCSSR1, CCSSR23, CCSSR29, CCSSR53, CCSSR59), HEX (CCSSR9, CCSSR55), and TAMRA (CCSSR17, CCSSR34). Capillary electrophoresis was performed on a 3130 × ABI Genetic Analyser (Applied Biosystems, Grand Island, NY, USA), and GeneMapper 4.0 (Applied Biosystems) was used to analyze the fragments and score the allele sizes. Negative controls (ddH_2_O) were included in each step of the analyses, to eliminate potential contamination.

**Table 2:** Markers and primers used in this study; all primers are from Ranathunge et al. (2009)

Marker name	Dye color	Primer sequence	Allele range (bp)	No. of alleles in our samples	H (SE)
CCSSR1	FAM	ACACGGCCTAGTTACGGTTG	106–226	23	0.409 (0.081)
		CCAATCGACTTTGGGAACAC			
CCSSR9	HEX	CAGATAATTTGGCCCGAAAA	172–190	7	0.448 (0.062)
		TTTTGCCTCGTATCCGTCTT			
CCSSR17	TAMRA	CACCTTACGGCTGCTAGTCC	159–187	12	0.417 (0.074)
		TGACGGTAAGCATGTCCTGA			
CCSSR23	FAM	GACGGTAAGAAACGGTGCAT	85–167	12	0.303 (0.070)
		TTTCTCTTCTCGCCTTCCTC			
CCSSR29	FAM	GCCTGGAGCGAAGATTGTTA	202–216	7	0.447 (0.062)
		GAGTGTTCTGCCCAAAGGAA			
CCSSR34	TAMRA	CGAATCGTCACCACGAACTA	169–221	17	0.372 (0.060)
		GGCAACTTCAAACGATGACA			
CCSSR53	FAM	TCGGCAACATACCTGAGACA	133–243	22	0.214 (0.052)
		GTCATGACGGTGTCGTGCT			
CCSSR55	HEX	CTGGGAAGATGAGCTGGATG	150–164	8	0.233 (0.060)
		GAGCAAACCCACCCACTTT			
CCSSR59	FAM	GTTTTTCCCTATCGCCCTGT	102–204	18	0.436 (0.070)
		CTTGAACAGCCGAGGTTAGG			

Dye colors: FAM = blue, HEX = green, TAMRA = yellow.

### Population genetics

The number of genotypes and genotypic diversity of the 13 populations were calculated separately. The haplotypes of each locus were also surveyed. To determine whether the number of loci used was sufficient to represent the genotypic diversity of the populations, we tested the relationship between the number of loci and genotype diversity. All tests were conducted using Multilocus1.3b (Agapow and Burt 2001).

Multilocus linkage disequilibrium was analyzed by two measures. First, the index of association (*I*_A_) in populations was examined using the Multilocus1.3b (Agapow and Burt 2001). *I*_A_ is a generalized measure of linkage disequilibrium and has an expected value of zero if the alleles at different loci are randomly associated with each other in the population, Significant associations between alleles at different loci would be expected in clonally reproducing populations (Xu 2006). The null hypothesis of complete panmixia was tested by comparing the observed value of the statistics set with 500 randomized data set (Agapow and Burt 2001). Second, the proportion of compatible pairs of loci (PrCP) was calculated using Multilocus1.3b. Briefly, two loci are compatible if it is possible to account for all the observed genotypes by mutations without inferring homoplasy (reversals, parallelisms, or convergences) or recombination; otherwise, the loci are incompatible. For example, for two loci with two alleles each, the loci are compatible, if no more than three genotypes are observed; if all four genotypes are observed, these two loci are phylogenetically incompatible. Phylogenetic incompatibility suggests recombination in the population. PrCP approaches a value of 1 for no recombination (Agapow and Burt 2001; Xu 2006; Liang et al. 2009; Chowdhary et al. 2011). *I*_A_ and PrCP were calculated based on the clone-corrected data (McDonald 1997).

### Population structure

The population structure of *C. truncatum* was analyzed in three ways. Firstly, a principal coordinates analysis (PCoA) was run by GenAlEx6 to calculate the Nei's unbiased genetic distance (Nei 1978) among all paired populations. Secondly, STRUCTURE 2.3.4 was also used to study the affiliation of individual isolates from sampling locations to specific clusters (*K*) and test for admixture (Pritchard et al. 2000; Hubisz et al. 2009). STRUCTURE implements a clustering algorithm based on a Bayesian Monte Carlo Markov Chain (MCMC) approach to assign individuals into *K* distinct populations. Using the admixture model, the number of clusters (*K*) was estimated, 10 replicated runs of *K* = 1–13 were carried out after a burn-in period of 100 000 generations followed by a run length of 100 000 generations. The number of genetically homogeneous clusters (*K*) was identified by following the method developed by Evanno et al. (2005) (Evanno et al. 2005).

Thirdly, analysis of molecular variance (amova) was used to calculate the genetic differentiation with the GenALEx6 (Peakall and Smouse 2006), which refers to the relative contribution among-and within-site components to the genetic variation.

The populations were divided into regions according to the results of the PCoA and STRUCTURE analysis, and the relative contributions within population genetic variation phiPT, between populations within regions phiRT, and between regions phiRT were calculated using GenAlEx6. The level of genetic differentiation among *C. truncatum* populations was also quantified using *R*_st_, which is a modified version of Wright's *F*_st_ and is used specifically for microsatellite data (Slatkin 1995; Xu 2006). Pairwise *R*_st_ was calculated and evaluated using a randomization test with 1000 interactions in GenAlEx6 (Peakall and Smouse 2006).

### Correlation between genetic variation and geographic separation

To examine whether genetic isolation was associated with geographic distance among *C. truncatum* populations, the relationship between the genetic distance and the geographic distance was determined with a Mantel test conducted with GenALEx6 (Peakall and Smouse 2006). The pairwise Nei's population genetic distances were calculated based on gene frequency differences between populations, and these distances were then compared to geographic distances between populations.

## Results

### Identification of *Colletotrichum truncatum*

In our study, all 266 isolates had falcate conidiophores, the color of colonies ranged from white to grayish dark on PDA, similar to characteristic of *C. truncatum* reported previously (Damm et al. 2009). The phylogenetic analysis based on sequences at six gene fragments showed that all 14 tested isolates clustered together with three known *C. truncatum* strains (ex-epitype CBS 151.35; and two other stains CBS136.30, CBS141.79, Figure S1). These results indicated that all our strains belonged to the same species.

### Genetic variation among loci and populations

The analyzed loci showed a high discriminating power among individual isolates. Based on results from randomizations, the nine loci were sufficient to achieve a high level of discrimination (Figure S2). The percentage of polymorphic loci was high in most populations. All the loci sequenced proved to be polymorphic in the total sample, the number of alleles per locus varied between 7 and 23, and gene diversity per locus ranged from 0.214 to 0.448 (Table2). Among the populations, a total of 148 multilocus genotypes were detected in 266 isolates based on the nine microsatellite loci. The number of genotypes for each population ranged from 3 to 32, and the northern populations had more genotypes (81) than southern populations (67). The total sample, the southern and northern populations, as well as most local populations, all showed high genotypic diversity with values ranging from 0.458 to 1 (Table3). Similarly, gene diversities (H) for the total sample, the southern and northern populations, as well as most local populations were high, with values ranging from 0.133 (LY) to 0.749 (total sample). The southern and northern populations had similar gene diversity (0.617–0.602). The number of private alleles for each population ranged from 2 (FX, LY, BJ, CC) to 11 (QY), and the southern populations had more private alleles (28) than northern populations (21) (Table3).

**Table 3:** Population genetic parameters for each of the 13 populations of *Colletotrichum truncatum* from chili peppers in China

Population	Percentage of polymorphic loci	No. of genotypes	Genotypic diversity	No. of alleles (SE)	H (SE)	Private alleles	PrCP	*I*_A_ (*P* value)
QY	100	23	0.866	4.111 (0.935)	0.341 (0.08)	11	0.472	1.292 (0.002)**
MM	100	10	0.923	3.333 (0.408)	0.471 (0.050)	4	0.722	0.043 (0.410)
YC	100	15	0.921	3.889 (0.754)	0.423 (0.089)	3	0.694	0.218 (0.128)
CQ	66.67	6	0.458	2.222 (0.364)	0.171 (0.058)	4	1	0.866 (0.026)*
WH	100	7	0.540	3.444 (0.377)	0.330 (0.032)	4	1	2.954 (0.002)**
FX	77.78	6	0.682	2.000 (0.289)	0.340 (0.074)	2	0.972	1.753 (0.006)**
Southern China-Total	100	67	0.953	9.889 (2.150)	0.617 (0.061)	28	0	0.688 (0.002)**
WC	100	32	0.963	6.333 (0.764)	0.599 (0.043)	4	0.25	1.365 (0.002)**
LY	33.33	3	0.511	1.444 (0.242)	0.133 (0.068)	2	—^d^	—
TJ	66.67	8	0.891	2.889 (0.696)	0.375 (0.111)	3	0.972	0.089 (0.332)
LF	88.89	14	0.889	3.333 (0.687)	0.356 (0.088)	5	0.944	1.364 (0.002)**
BJ	77.78	13	0.877	3.333 (0.726)	0.302 (0.072)	2	0.972	1.698 (0.002)**
XC	66.67	8	0.700	2.556 (0.444)	0.294 (0.087)	3	0.944	0.214 (0.188)
CC	100	5	1	3.222 (0.324)	0.604 (0.048)	2	0.944	0.257 (0.266)
Northern China-Total	100	81	0.981	10.111 (1.006)	0.602 (0.054)	21	0.056	0.994 (0.002)**
Total	100	148	0.983	13.667 (2.055)	0.749 (0.035)	49	0	1.228 (0.002)**

H, gene diversity; PrCP, proportion of phylogenetically compatible pairs of loci; *I*_A_, index of association; —, not analyzed because of small sample size.

* *P* < 0.05.

** *P* < 0.01.

### Allelic associations

The index of association (*I*_A_) was calculated for each population and the entire sample. The *I*_A_ values for seven populations and for the total samples were significantly higher than the simulated data sets obtained assuming panmixia (Table3). The remaining populations did not show significant allelic association among loci. The PrCP showed that most populations and the total samples had evidence of phylogenetic incompatibility. When the *I*_A_ and the PrCP results were combined, there was unambiguous evidence for substantial recombination and sexual reproduction in most populations. Our results indicate that both sexual and asexual reproductions have occurred in all populations and that some populations were predominantly clonal. For both the southern and the northern regional populations, the *I*_A_ values did not support random recombination, but there was unambiguous evidence for recombination in both populations based on PrCP values (0/0.05).

### Population structure

**Figure 2:**
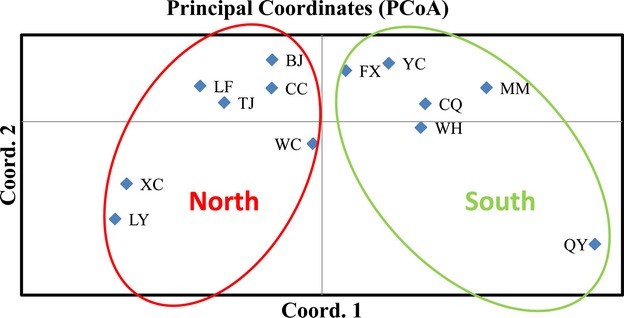
Principal coordinate analysis of 13 populations of *Colletotrichum truncatum* in China based on Nei's genetic distance using GenALEX. The left circle contains all of the northern populations, and the right circle contains all of the southern populations.

PCoA revealed two clusters of *C. truncatum* isolates; axes 1 and 2 of the PCoA accounted for 74.48% and 17.96% of the total genetic variation (Fig.2). PCoA also indicated that the six populations from southern China were clustered in one group in the right quadrants of the first principal coordinate, while the remaining seven populations from northern China clustered in the left quadrants (Fig.2).

A similar clustering pattern was obtained by STRUCTURE analyses. The highest likelihood values and the mode of the distribution of the *ΔK* index were all observed for *K* = 2, significantly higher than other clusters. The *K* = 2 separated the southern populations from the northern populations (Fig.1B). Among the populations, WC showed a mixed ancestry, while CC, WH, MM, and TJ populations showed low levels of admixtures. In the deltaK analysis, there was a small secondary peak at *K* = 7, likely caused by these mixed populations (Fig.1B and Figure S3).

The amova results showed that 38%, 28%, and 34% of the genetic variation could be attributed to variations among regions (north and south), among populations within regions, and among individual isolates within populations respectively. All three sources of variation were significant (*P* < 0.01) (Table4).

**Table 4:** Analysis of molecular variance (amova) within and among 13 *Colletotrichum truncatum* populations in China

Source*	df	SS	MS	Estimated. Variance.	Percentage	Stat	Value	*P*
Among regions	1	154094.307	154094.307	1001.802	38	PhiRT	0.377	0.001
Among populations	11	168122.967	15283.906	750.029	28	PhiPR	0.453	0.001
Within populations	253	229579.057	907.427	907.427	34	PhiPT	0.659	0.001
Total	265	551796.331		2659.258				

* There were two regions (northern China and southern China), 13 populations, and 204 isolates; df, degree of freedom; SS, sum of squared observations; MS, mean of squared observations; PhiRT, proportion of the total genetic variance that are between regions; PhiPR, proportion of the total genetic variance that are among populations within a region; PhiPT, proportion of the total genetic variance that are among individuals within a population.

**Figure 3:**
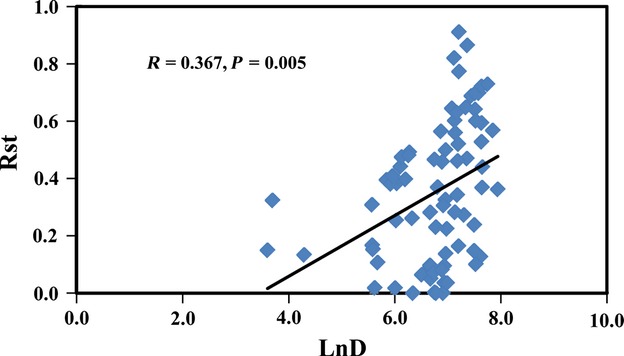
Correlation between genetic differentiation *R*_st_ and geographic distance (ln) among *Colletotrichum truncatum* populations in China according to the Mantel test (Rxy = 0.367, *P* = 0.005).

The *R*_st_ value was the lowest between populations FX and YC and was the highest between populations CQ and LY. In most cases, *R*_st_ values were consistently higher between pairs of southern and northern populations than between pairs from within the south or within the north. The Mantel test showed that geographic distance (Ln) and genetic differentiation (*R*_st_) among geographic populations were positively correlated (Fig.3; *P* = 0.005), with a correlation coefficient of 0.367.

### Host germplasm diversity center and pathogen diversity

The QY population, which came from a chili pepper seed-breeding center in Qingyuan in Guangdong Province in southern China, harbored the most private alleles (11). After excluding the influence of sample size, we found that the number of private alleles in QY population was still significantly greater than expected (Figure S4) if we assume a linear relationship between sample size and number of private alleles. However, the *I*_A_ value of QY population did not support random recombination, but the PrCP (0.472) was lower than most other populations, consistent with the host diversity playing a role in pathogen genotype diversity. Other parameters such as the number of genotypes and the total number of alleles also showed a similar pattern. The only population showing more genotypes, higher allelic richness, and lower linkage disequilibrium than the QY population was the WC population. However, the WC population had a lower than expected number of private alleles.

## Discussion

In this study, we analyzed the population genetics of *C. truncatum* in China and found evidence of recombination in *C. truncatum* populations on chili in China. We also uncovered that the Chinese *C. truncatum* populations could be clustered into two distinct genetic groups, which correspond to the geographic boundary between southern and northern China. Below, we discuss the relevance of our results in the management and prevention of anthracnose in chili peppers.

### Recombination in *C. truncatum* populations

Our study revealed high genetic diversities in *C. truncatum* populations on chili peppers in China and suggested that substantial sexual recombination occurred in these populations. Similarly, isolates of *C. gloeosporioides* from strawberry revealed a low level of linkage disequilibrium as would be expected in sexually recombining populations (Ureña-Padilla et al. 2002). These findings contrast those reported by several others (Sicard et al. 1997; Rosewich et al. 1998; Chen et al. 2002), which indicated that *C. graminicola* and *C. lindemuthianum* were clonal and exhibited limited sexual recombination and low genetic diversity. The observed population genetic differences among *Colletotrichum* species are not surprising given the differences in the type of markers and the scale of sampling (Leung et al. 1993). In addition, the reproductive mode of a fungus can vary in space and time (Taylor et al. 1999). And for other fungi, similar results were also observed, such as *Candida albicans*, which showed both clonality and recombination, even though a complete sexuality stage is not known to exist in this fungus (Gräser et al. 1996).

Evidence for frequent recombination in natural populations of *C. truncatum* suggests that a sexual teleomorph likely exists for this organism in China. However, at present, we cannot exclude the possibility that parasexual recombination could also contribute to the observed linkage equilibrium and phylogenetic incompatibility (Sicard et al. 1997). In most microscopic fungi, their sexual cycles can be difficult to observe in nature (Calo et al. 2013), and inferences about the potential sexual cycle have largely relied on the analyses of gene and genotype frequencies in natural populations. In some organisms such as *C. lindemuthianum*, a sexual cycle has been described *in vitro*, but there was a low viability for the sexual ascospores (Bryson et al. 1992).

### Population structure and differentiation

The *C. truncatum* populations from chili peppers in China clustered into two distinct genetic groups (Figs1 and 2). Interestingly, the clusters corresponded to the geographic boundary of southern and northern China. This genetic differentiation is probably caused by differences in geography. The south–north boundary of China is the Qinling Mountain range, which is the boundary separating the subtropical zone from the warm temperate zone, the humid from the semi-humid climate, and the rivers without icy cover from those with at least a short icy cover. Consistent with the hypothesis of a geographic barrier to gene flow playing a large role in *C. truncatum* populations on Chinese chili peppers, the WC population was closest to Qinling Mountains and it showed a mixed ancestry (Fig.1).

A similar geography-based separation of *C. truncatum* populations has been reported for samples from two states in Malaysia (Mahmodi et al. 2013; Sharma et al. 2014). Geographic differentiation has also been reported between the eastern and western African populations of another anthracnose pathogen, *Colletotrichum kahawae* (Silva et al. 2012). All the isolates from eastern Africa were clustered together, and isolates from two western African countries (Angola and Cameroon) were more closely related. Western and eastern African populations were separated by extensive lowland areas, which might not have been suitable for the pathogen nor its hosts, thus representing an effective barrier for gene flow (Silva et al. 2012). The significant geographic contribution to the overall genetic differences in *C. truncatum* was also supported by the positive correlation between genetic distance and geographic distance among strains and populations in our samples.

While the major differentiation was between samples separated by the south–north geographic line (Table3), significant differences in gene frequencies were also found among samples within both the southern and the northern regions (Table5). The *R*_st_ values were overall very high and two possibilities might have contributed to this. First, *C. truncatum* is seedborne and can also be dispersed in short distance by rain from contaminated soil and infected host debris. If seeds, soil, and plant debris were dispersed only through short distances, geographic populations separated by long distances would be genetically differentiated. Second, *C. truncatum* has many hosts including other crops and weeds (McLean and Roy 1991). Given that weeds and other crops are common around chili pepper fields in China, host shifts may be frequent. This could increase the probability that *C. truncatum* on other nearby hosts could mate, which could increase genetic variation within and among populations. On the other hand, host shifts could also drive evolution of pathogen and lead to ecological speciation (Giraud et al. 2010; Raffaele et al. 2010; Silva et al. 2012). Although generally high, *R*_st_ values were low between some *C. truncatum* populations. A substantial range in *R*_st_ values has also been reported for other fungi. In the case of *Laccaria amethystine*, for example, the *F*_*s*t_ value was as high as 0.516 between Japanese and European populations but was only 0.041 among European populations (Vincenot et al. 2012).

**Table 5:** Pairwise *R*_st_ values between 13 populations of *Colletotrichum truncatum* from China

Population	QY	MM	YC	CQ	WH	FX	WC	LY	TJ	LF	BJ	XC
MM	0.308**											
YC	0.491**	0.097										
CQ	0.370**	0.035	0.064									
WH	0.282**	0.036	0.018	−0.009								
FX	0.520**	0.273*	−0.018	0.441**	0.050							
WC	0.469**	0.238**	0.137**	0.094	0.094	0.071						
LY	0.688**	0.698**	0.645**	0.911*	0.565**	0.820*	0.253*					
TJ	0.641**	0.528**	0.342**	0.559**	0.326**	0.306*	0.167*	0.383				
LF	0.697**	0.594**	0.461**	0.602**	0.499**	0.458**	0.153**	0.475**	0.324**			
BJ	0.601**	0.368**	0.164*	0.282*	0.224*	0.081	0.107*	0.397*	0.133*	0.150*		
XC	0.721**	0.729**	0.648**	0.864**	0.630**	0.773**	0.261**	0.018	0.395*	0.381**	0.408**	
CC	0.568**	0.362*	0.126	0.440*	0.148	0.101	0.035	0.466*	0.003	0.230**	−0.053	0.480**

* *P* < 0.05.

** *P* < 0.01.

### Genetic variation and population structure among the southern Chinese, the northern Chinese, and the Indian populations

Different from previous studies of *C. truncatum* where different markers were used, a recent study of Indian strains used the same molecular markers that we used here (Sharma et al. 2014). In that study, the majority of the strains came from Bengaluru (13.05N, 77.58E) in southern India. The genotype information at shared loci allowed us to compare the Indian *C. truncatum* population with those from southern and northern China. Our PCoA result showed that the Indian population was distinctly different from the Chinese populations, consistent with geography playing a significant role in the structure of global *C. truncatum* populations. Among the three *C. truncatum* populations, the Indian and the southern Chinese populations were more similar to each other than either was to the northern Chinese population (Figure S5). Such a pattern might reflect the similarity in climate conditions between southern China and southern India. Similar to the results on the Chinese populations, additional analyses of the Indian samples showed evidence of recombination but not random mating (Table S3). Interestingly, while the Indian population had a genotypic diversity similar to our samples, it had more private alleles and higher gene diversity than our samples (Table S3). At present, the reasons for such differences are unknown, but they may reflect the sample types analyzed. All our samples were from Chili peppers. However, the Indian samples came from a diversity of host plant species (Sharma et al. 2014).

### Host germplasm diversity center and pathogen diversity

Among the populations, the QY population showed a very high *R*_st_ value when compared with other populations (Table5). It also had a greater than expected number of private alleles (Figure S4). In contrast, the WC population had fewer private alleles than expected (Figure S4). In many agricultural crops, monocultures have shown to be more susceptible to infectious diseases, likely as a result of selection for specific virulent genotype and the rapid expansion and dispersal of such genotype (Zhu et al. 2000). As a result, continued monoculturing would select for reduced pathogen genotypes that are particularly targeted for specific host genotypes. In contrast, a high host genotype diversity would likely be correlated with high pathogen diversity. Among all the population genetic parameters, only the number of private alleles was found high for the QY population and low for the WC population. While this result is consistent with the effect of high host diversity (QY population) and long-term continued monocultures (for the WC population), whether the number of private alleles at individual populations reflects the differences in host genotype diversity remains unknown. To address this question, long-term surveys of genotypes of both pathogen and host plants are needed.

### Clonal dispersal

Although there was significant genetic differentiation among populations within both the southern and the northern regions, clonal dispersal was observed between adjacent geographic areas (Table S2). Here, the maximum distance of clonal dispersal was 75 km, between BJ and LF populations (Table S2). The clonal dispersal could be achieved through chili pepper seed-dispersal, windborne, or soilborne (Rosewich et al. 1998; McDonald and Linde 2002). Similarly, clonal dispersal of *C. graminicola* was also reported to be significant over large geographic scale (between Georgia, Honduras, and Zambia), likely through both windborne and seedborne due to the movement of contaminated seeds.

### Implications for disease management

Statistically significant genetic isolation by geographic distance was found in our analyses. Such a result suggests that geography can act as a partial barrier to prevent the homogenization of the pathogen populations in China. However, as has been found in many fungi, human activities such as international travel and trade can be a significant factor in facilitating fungal dispersals (Khankhet et al. 2014), especially for pathogens associated with vegetables and crops (Harlan 1976; Thresh 1982; Anderson et al. 2004;). Thus, we believe strict quarantine measures should be taken to avoid its dispersal.

## Conclusion

In this study, we analyzed the population genetics of *C. truncatum* in China. We found a high level of genetic variation and evidence for recombination in *C. truncatum* populations on chili in China. We revealed that the Chinese *C. truncatum* populations were clustered into two distinct genetic groups, one in southern China and the other in northern China. Although the north/south divide contributed the most to the observed genetic variation, within both the southern and the northern regions, subtle genetic differences were still identified among local populations. Whether other plant fungal pathogens follow a similar geographic pattern remains to be examined. The knowledge of population structure of *C. truncatum* could have a significant impact on pathologists and breeders to screen chili pepper germplasms for new sources of resistance genes. *C. truncatum* is worldwide distributed and has many hosts. The relationship among global geographic populations of *C. truncatum* and from different hosts remains to be explored.

## Data archiving statement

Data for this study are available in the supporting information online.

## Supporting Information

**Figure S1.** Phylogram generated with maximum likelihood (ML) analysis based on the Tamura-Nei+Gamma model, with combined ACT, GAPDH, ITS, TUB2, CHS-1, and His3 sequences. **Figure S2.** Relationship between the number of loci and genotype diversity for *Colletotrichum truncatum* in China. **Figure S3.** Evanno plot derived from STRUCTURE HARVESTER for detecting number of genetic clusters. **Figure S4.** The relationship between numbers of isolates and private alleles. **Figure S5.** Principal coordinate analysis of 3 populations of *Colletotrichum* truncatum based on Nei's genetic distance using GenALEX.

**Table S1.** Information about strains of *Colletotrichum* analyzed in this paper for sequence-based species identifications. GenBank accessions for individual sequences are shown.

**Table S2.** Number and clone range of multilocus genotypes (MLG) of *C. truncatum* based on analysis of nine microsatellite loci.

**Table S3.** Genotypic diversity, number of genotypes, proportion of compatible pairs of loci (PrCP), and Index of association (I_A_), h (gene diversity), allelic richness, percentage of polymorphic loci, number of private alleles within the Southern Chinese, Northern Chinese, and Indian populations of *Colletotrichum truncatum*.

**Table S4.** Origin and microsatellite profiles for 266 isolates (strains in grey were not included in the clone corrected data).
