# Supplementary material for: Genetic differentiation and recombination among geographic populations of the fungal pathogen Colletotrichum truncatum from chili peppers in China
Source: Evol Appl. 2014 Dec 13;8(1):108–18. doi: 10.1111/eva.12233 (PMC4310585; doi:10.1111/eva.12233)
Supplement: Supplementary file 1 [file eva0008-0108-sd1.pdf]

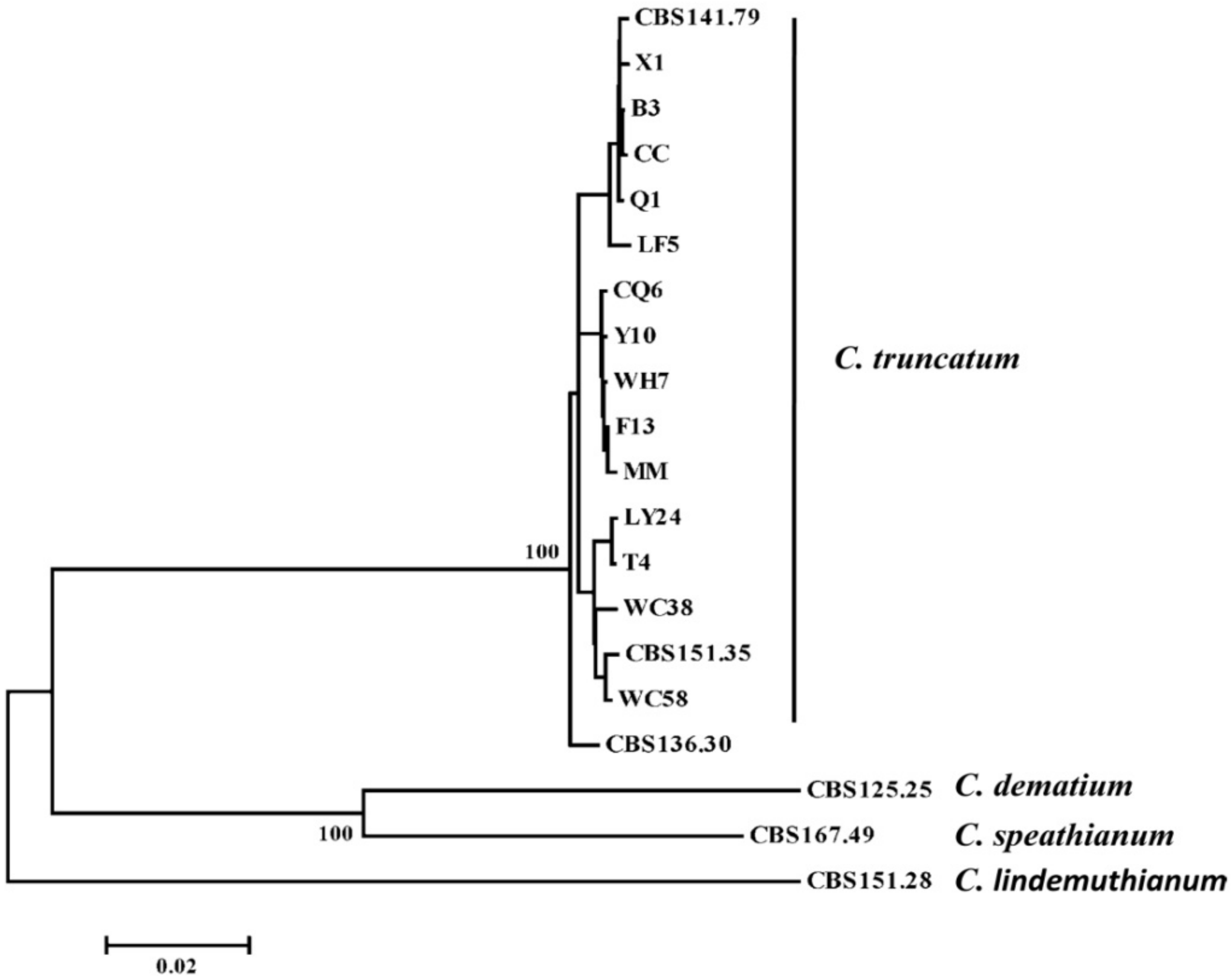


**Fig. S1**. Phylogram generated with maximum likelihood (ML) analysis based on the Tamura-Nei+Gamma model, with combined ACT, GAPDH, ITS, TUB2, CHS-1, and His3 sequences. *C. dematium and C. speathianum are closely related species to C. truncatum.*

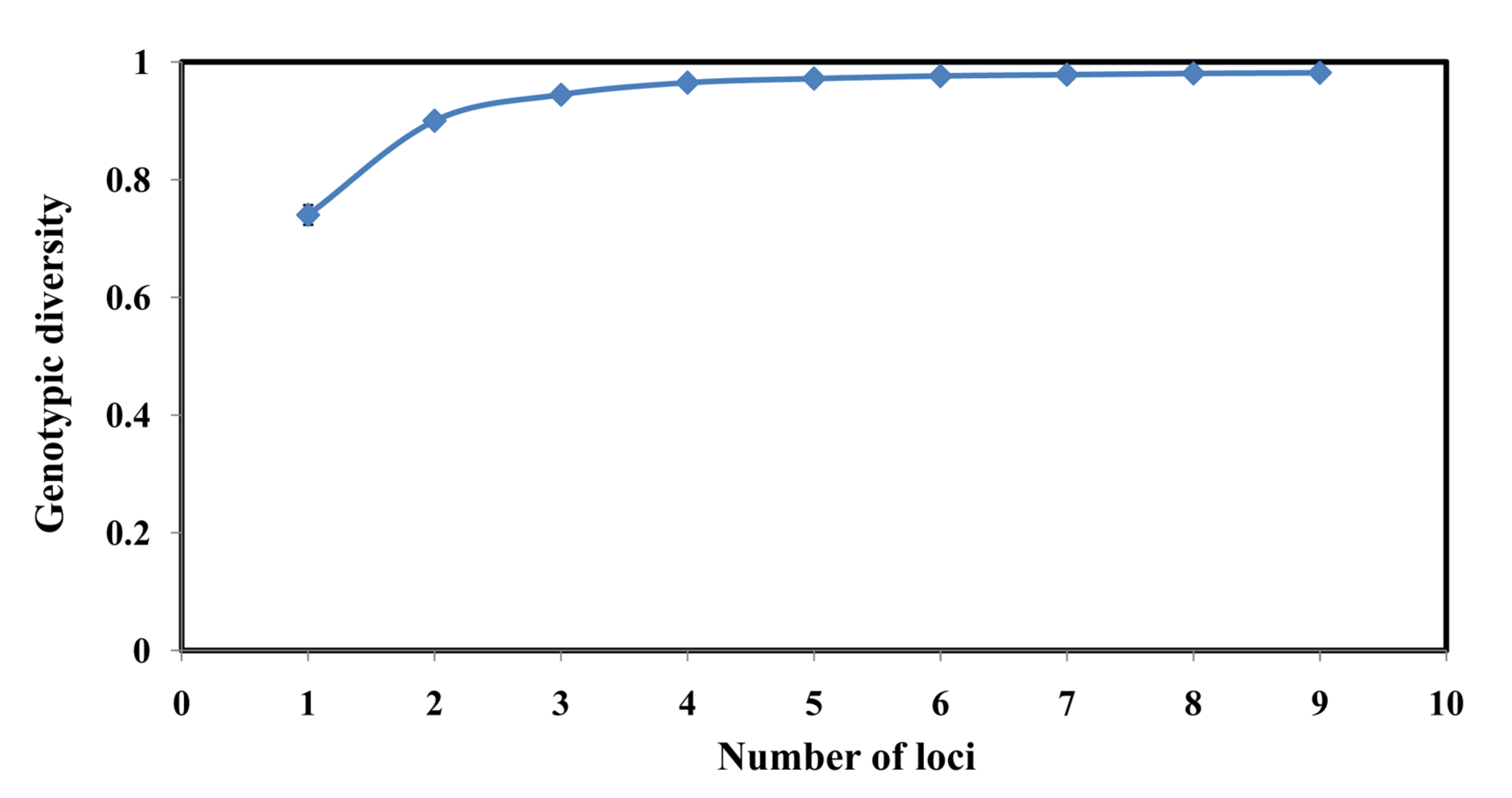


**Fig. S2** Relationship between the number of loci and genotype diversity for *Colletotrichum truncatum* in China.

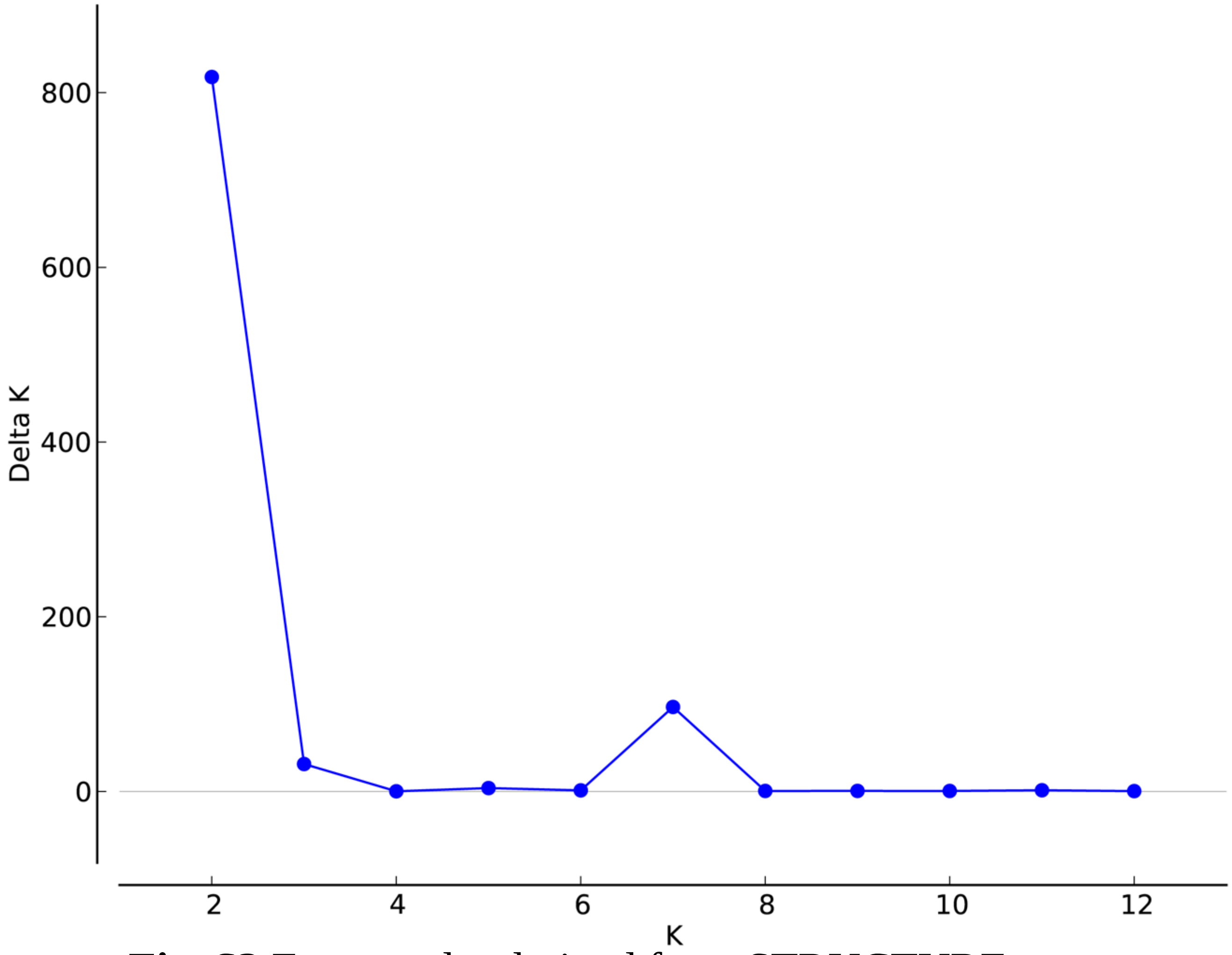


**Fig. S3** Evanno plot derived from STRUCTURE HARVESTER for detecting number of genetic clusters.

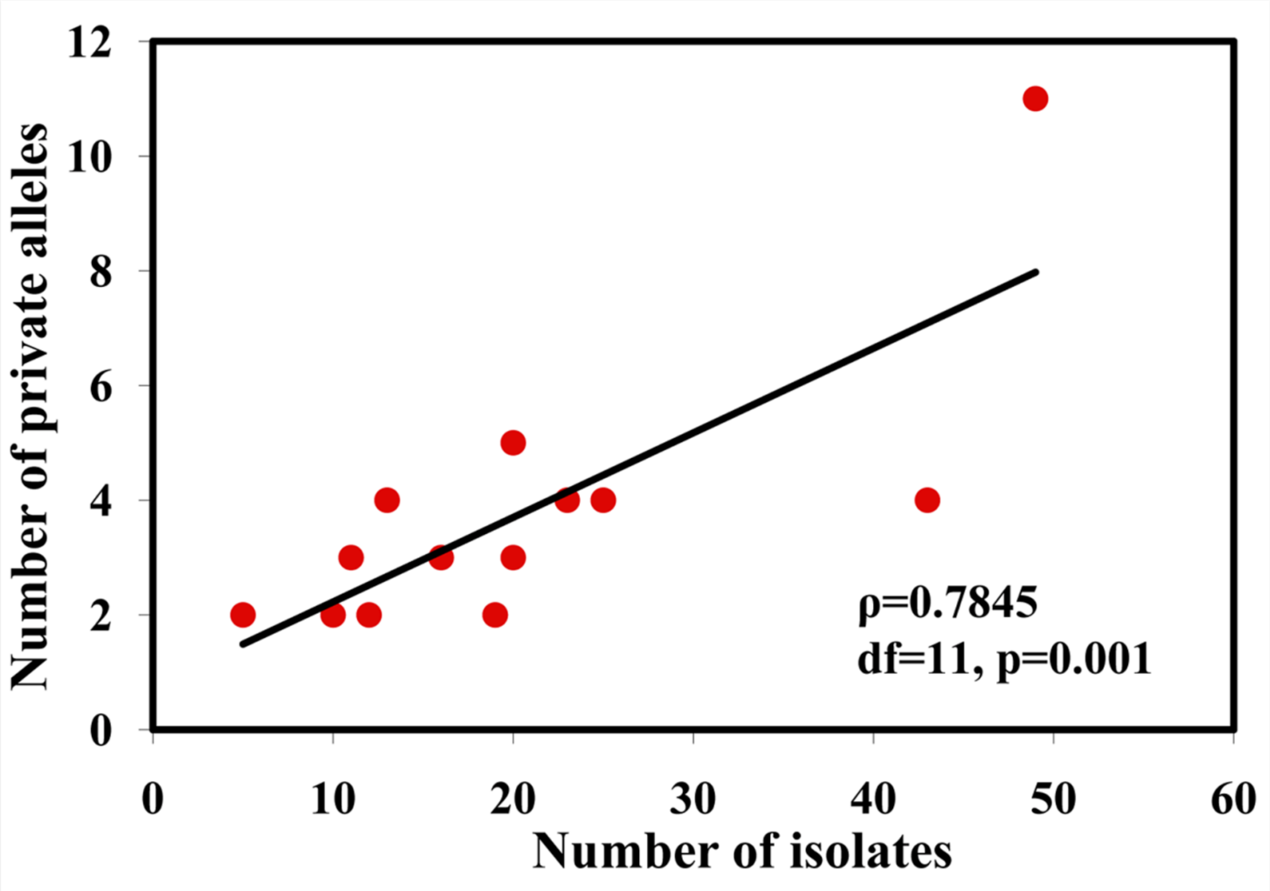


**Fig. S4** The relationship between numbers of isolates and private alleles. Based on the regression, the QY population (with 11 private alleles among 49 isolates) had more private alleles than expected while the WC population (with four private alleles among 43 isolates) had fewer private alleles than expected.

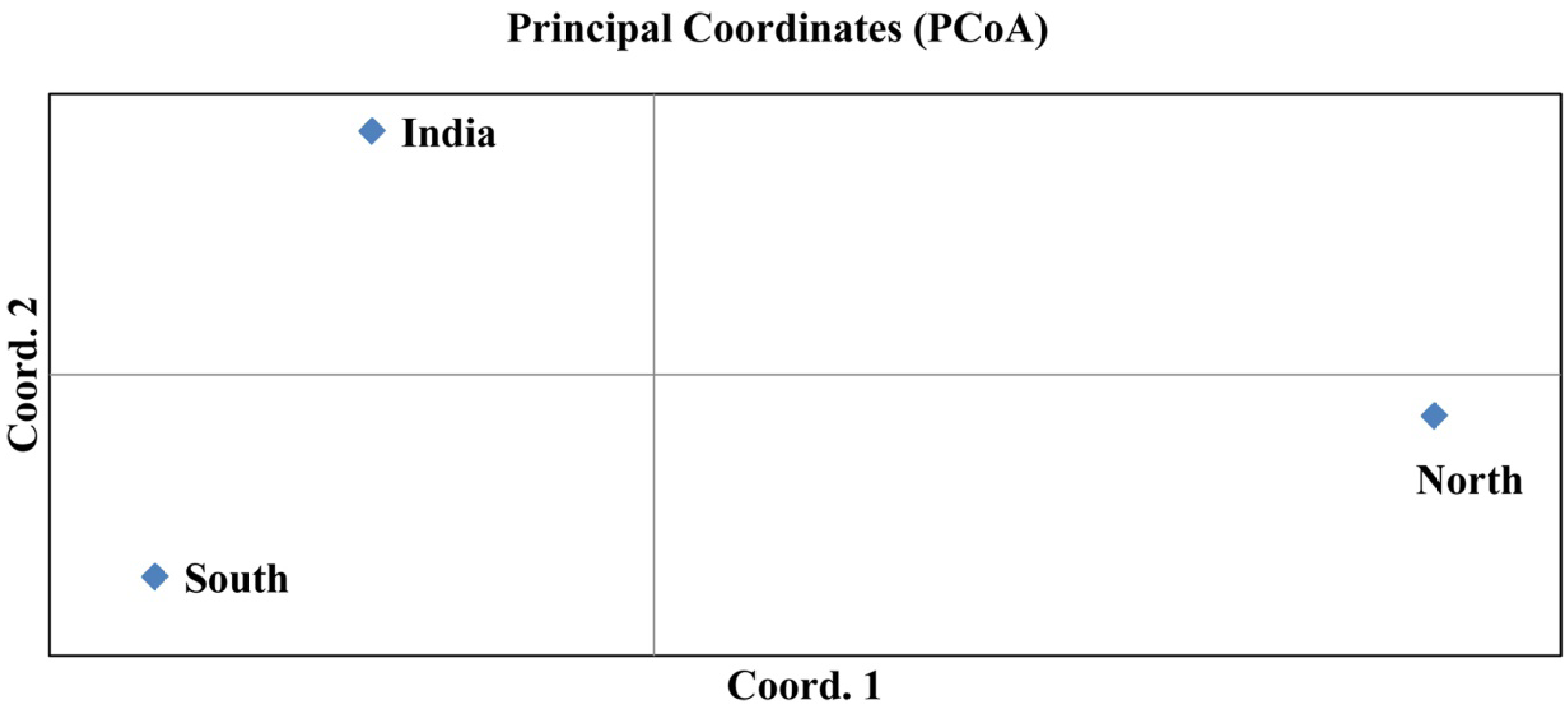


**Fig. S5.** Principal coordinate analysis of 3 populations of *Colletotrichum truncatum based on Nei's genetic distance using GenALEX. South and North stand for the* southern and northern populations of China, respectively, and India refers to India population. (Axes1 and 2 of the PCoA accounted for 77.94% and 22.06% of the total genetic variation )
