# Supplementary material for: Genetic differentiation and recombination among geographic populations of the fungal pathogen Colletotrichum truncatum from chili peppers in China
Source: Evol Appl. 2014 Dec 13;8(1):108–18. doi: 10.1111/eva.12233 (PMC4310585; doi:10.1111/eva.12233)
Supplement: Supplementary file 2 [file eva0008-0108-sd2.docx]

**Table S1.** Information about strains of *Colletotrichum* analyzed in this paper for sequence-based species identifications. GenBank accessions for individual sequences are shown.

| **Species** | **Accession number** | **Host** | **Country** | **Genbank accessions** | | | | | |
| --- | --- | --- | --- | --- | --- | --- | --- | --- | --- |
|  |  |  |  | **ITS** | **ACT** | **Tub2** | **CHS-1** | **GAPDH** | **HIS3** |
| *C. truncatum* | CBS 151.35* | *Phaseolus lunatus* | USA | GU227862 | GU227960 | GU228156 | GU228352 | GU228254 | GU228058 |
| *C. truncatum* | CBS141.79 | *Stylosanthes hamata* | Australia | GU227873 | GU227971 | GU228167 | GU228363 | GU228265 | GU228069 |
| *C. truncatum* | CBS136.30 | *Crotalaria juncea* | Trinidad and Tobago | GU227876 | GU227974 | GU228170 | GU228366 | GU228268 | GU228072 |
| ***C. truncatum*** | **B3** | ***Capsicum annuum*** | **China** | **KJ482232** | **KJ482172** | **KJ482157** | **KJ482187** | **KJ482202** | **KJ482217** |
| ***C. truncatum*** | **CC** | ***Capsicum annuum*** | **China** | **KJ482233** | **KJ482173** | **KJ482158** | **KJ482188** | **KJ482203** | **KJ482218** |
| ***C. truncatum*** | **CQ6** | ***Capsicum annuum*** | **China** | **KJ482234** | **KJ482174** | **KJ482159** | **KJ482189** | **KJ482204** | **KJ482219** |
| ***C. truncatum*** | **F13** | ***Capsicum annuum*** | **China** | **KJ482235** | **KJ482175** | **KJ482160** | **KJ482190** | **KJ482205** | **KJ482220** |
| ***C. truncatum*** | **LF5** | ***Capsicum annuum*** | **China** | **KJ482236** | **KJ482176** | **KJ482161** | **KJ482191** | **KJ482206** | **KJ482221** |
| ***C. truncatum*** | **LY24** | ***Capsicum annuum*** | **China** | **KJ482237** | **KJ482177** | **KJ482162** | **KJ482192** | **KJ482207** | **KJ482222** |
| ***C. truncatum*** | **MM** | ***Capsicum annuum*** | **China** | **KJ482238** | **KJ482178** | **KJ482163** | **KJ482193** | **KJ482208** | **KJ482223** |
| ***C .truncatum*** | **QY1** | ***Capsicum annuum*** | **China** | **KJ482239** | **KJ482179** | **KJ482164** | **KJ482194** | **KJ482209** | **KJ482224** |
| ***C. truncatum*** | **T4** | ***Capsicum annuum*** | **China** | **KJ482241** | **KJ482181** | **KJ482166** | **KJ482196** | **KJ482211** | **KJ482226** |
| ***C.truncatum*** | **WC38** | ***Capsicum annuum*** | **China** | **KJ482242** | **KJ482182** | **KJ482167** | **KJ482197** | **KJ482212** | **KJ482227** |
| ***C. truncatum*** | **WC58** | ***Capsicum annuum*** | **China** | **KJ482243** | **KJ482183** | **KJ482168** | **KJ482198** | **KJ482213** | **KJ482228** |
| ***C. truncatum*** | **WH7** | ***Capsicum annuum*** | **China** | **KJ482244** | **KJ482184** | **KJ482169** | **KJ482199** | **KJ482214** | **KJ482229** |
| ***C. truncatum*** | **XC1** | ***Capsicum annuum*** | **China** | **KJ482245** | **KJ482185** | **KJ482170** | **KJ482200** | **KJ482215** | **KJ482230** |
| ***C. truncatum*** | **Y10** | ***Capsicum annuum*** | **China** | **KJ482246** | **KJ482186** | **KJ482171** | **KJ482201** | **KJ482216** | **KJ482231** |
| *C. dematium* | CBS 125.25* | *Eryngium campestre* | France | GU227819 | GU227917 | GU228113 | GU228309 | GU228211 | GU228015 |
| *C. spaethianum* | CBS167.49 | *Hosta sieboldiana* | Germany | GU227807 | GU227905 | GU228101 | GU228297 | GU228199 | GU228003 |
| *C. lindemuthianum* | CBS151.28 | *Phaseolus vulgaris* | UK | GU227800 | GU227898 | GU228094 | GU228290 | GU228192 | GU227996 |

^a^CBS Culture collection of the Centraalbureau voor Schimmelcultures, Fungal Biodiversity Centre, Utrecht, The Netherlands; Strains and sequences generated in this paper are in bold.
