# Supplementary material for: Genetic differentiation and recombination among geographic populations of the fungal pathogen Colletotrichum truncatum from chili peppers in China
Source: Evol Appl. 2014 Dec 13;8(1):108–18. doi: 10.1111/eva.12233 (PMC4310585; doi:10.1111/eva.12233)
Supplement: Supplementary file 3 [file eva0008-0108-sd3.docx]

**Table S2.** Number and clone range of multilocus genotypes (MLG) of *C. truncatum* based on analysis of nine microsatellite loci.

| Repeated MLG | Number of isolates | Population | Clone range (km) |
| --- | --- | --- | --- |
|  |  |  |  |
| MLG1 | 18 | QY | 1 |
| MLG2 | 3 | QY | 1 |
| MLG3 | 3 | QY | 1 |
| MLG4 | 4 | QY | 1 |
| MLG5 | 4 | MM | 3 |
| MLG6 | 5 | YC | 1 |
| MLG7 | 2 | CQ | 3 |
| MLG8 | 17 | CQ | 3 |
| MLG9 | 2 | WH | 3 |
| MLG10 | 17 | WH | 3 |
| MLG11 | 7 | FX | 1 |
| MLG12 | 2 | WC | 2 |
| MLG13 | 8 | WC | 2 |
| MLG14 | 2 | WC | 2 |
| MLG15 | 3 | WC | 2 |
| MLG16 | 2 | LY | 1 |
| MLG17 | 7 | LY | 1 |
| MLG18 | 4 | TJ | 1 |
| MLG19 | 8 | LF/BJ | 75 |
| MLG20 | 2 | LF/BJ | 75 |
| MLG21 | 7 | BJ | 2 |
| MLG22 | 9 | XC | 2 |
