# Supplementary material for: Genetic differentiation and recombination among geographic populations of the fungal pathogen Colletotrichum truncatum from chili peppers in China
Source: Evol Appl. 2014 Dec 13;8(1):108–18. doi: 10.1111/eva.12233 (PMC4310585; doi:10.1111/eva.12233)
Supplement: Supplementary file 4 [file eva0008-0108-sd4.docx]

**Table S3.** Genotypic diversity, number of genotypes, proportion of compatible pairs of loci (PrCP), and Index of association (I_A_), h (gene diversity), allelic richness, percentage of polymorphic loci, number of private alleles within the Southern Chinese, Northern Chinese, and Indian populations of *Colletotrichum truncatum*.

| Location | N | Genotype diversity | Number of genotypes | | PrCP^a^ | I_A_ (P value)^b^ | H^c^ | No. of Alleles | Percentage of polymorphic loci | No. Private  alleles | |
| --- | --- | --- | --- | --- | --- | --- | --- | --- | --- | --- | --- |
| Southern China | 109 | 0.953 | 67 | 0 | | 0.688(0.002) ^**^ | 0.617 | 9.889 | 1 | 1.213 |  |
| Northern China | 95 | 0.981 | 81 | 0.056 | | 0.994(0.002) ^**^ | 0.602 | 10.111 | 1 | 0.745 |  |
| India | 99 | 1 | 99 | 0 | | 0.466(0.002) ^**^ | 0.94 | 29.111 | 1 | 1.908 |  |

^a^, PrCP, proportion of phylogenetically-compatible pairs of loci, ^b^, I_A_, index of association; ^c^, H, gene diversity; **p<0.01
