# Supplementary material for: Genetic differentiation and recombination among geographic populations of the fungal pathogen Colletotrichum truncatum from chili peppers in China
Source: Evol Appl. 2014 Dec 13;8(1):108–18. doi: 10.1111/eva.12233 (PMC4310585; doi:10.1111/eva.12233)
Supplement: Supplementary file 5 [file eva0008-0108-sd5.docx]

**Table S4** Origin and microsatellite profiles for 266 isolates (strains in grey were not included in the clone corrected data)

| Isolates | Locations | Loci | | | | | | | | |
| --- | --- | --- | --- | --- | --- | --- | --- | --- | --- | --- |
|  |  | CCSSR1 | CCSSR53 | CCSSR59 | CCSSR9 | CCSSR17 | CCSSR23 | CCSSR55 | CCSSR29 | CCSSR34 |
| QY1 | Qingyuan | 200 | 183 | 174 | 187 | 173 | 103 | 162 | 212 | 189 |
| QY2 |  | 200 | 145 | 168 | 184 | 173 | 103 | 158 | 212 | 177 |
| QY3 |  | 204 | 163 | 174 | 187 | 173 | 105 | 158 | 212 | 187 |
| QY4 |  | 200 | 183 | 174 | 187 | 173 | 105 | 162 | 212 | 189 |
| QY5 |  | 206 | 161 | 174 | 187 | 173 | 105 | 162 | 202 | 189 |
| QY6 |  | 200 | 183 | 174 | 187 | 173 | 105 | 162 | 212 | 189 |
| QY7 |  | 200 | 183 | 174 | 187 | 173 | 105 | 162 | 212 | 189 |
| QY8 |  | 130 | 199 | 144 | 187 | 173 | 103 | 158 | 212 | 185 |
| QY9 |  | 204 | 145 | 174 | 187 | 187 | 105 | 162 | 202 | 189 |
| QY10 |  | 204 | 161 | 174 | 187 | 173 | 105 | 158 | 212 | 187 |
| QY11 |  | 200 | 183 | 174 | 187 | 173 | 105 | 162 | 212 | 189 |
| QY12 |  | 200 | 183 | 174 | 187 | 173 | 105 | 162 | 212 | 189 |
| QY13 |  | 206 | 181 | 174 | 187 | 173 | 103 | 162 | 202 | 189 |
| QY14 |  | 200 | 183 | 174 | 187 | 173 | 105 | 162 | 212 | 189 |
| QY15 |  | 206 | 183 | 174 | 187 | 173 | 105 | 158 | 212 | 187 |
| QY16 |  | 200 | 183 | 174 | 187 | 173 | 105 | 162 | 212 | 189 |
| QY17 |  | 200 | 183 | 174 | 187 | 173 | 105 | 162 | 212 | 189 |
| QY18 |  | 204 | 161 | 174 | 187 | 173 | 105 | 162 | 202 | 189 |
| QY19 |  | 204 | 187 | 174 | 187 | 173 | 105 | 162 | 202 | 189 |
| QY20 |  | 204 | 183 | 174 | 187 | 173 | 105 | 162 | 202 | 189 |
| QY21 |  | 200 | 183 | 174 | 187 | 173 | 105 | 162 | 212 | 189 |
| QY22 |  | 200 | 183 | 174 | 187 | 173 | 105 | 162 | 212 | 189 |
| QY23 |  | 204 | 145 | 174 | 187 | 173 | 105 | 162 | 202 | 189 |
| QY24 |  | 204 | 161 | 174 | 187 | 173 | 105 | 162 | 202 | 189 |
| QY25 |  | 200 | 183 | 174 | 187 | 173 | 103 | 162 | 212 | 189 |
| QY26 |  | 206 | 145 | 174 | 187 | 173 | 105 | 162 | 202 | 189 |
| QY27 |  | 206 | 161 | 174 | 187 | 173 | 105 | 162 | 202 | 189 |
| QY28 |  | 200 | 183 | 174 | 187 | 173 | 105 | 162 | 212 | 189 |
| QY29 |  | 204 | 145 | 174 | 187 | 173 | 103 | 162 | 202 | 189 |
| QY30 |  | 204 | 161 | 174 | 187 | 173 | 105 | 162 | 202 | 189 |
| QY31 |  | 206 | 145 | 174 | 187 | 173 | 105 | 162 | 202 | 189 |
| QY32 |  | 206 | 179 | 129 | 187 | 173 | 105 | 158 | 212 | 187 |
| QY33 |  | 200 | 183 | 174 | 187 | 173 | 105 | 162 | 212 | 189 |
| QY34 |  | 200 | 183 | 174 | 187 | 173 | 105 | 162 | 212 | 189 |
| QY35 |  | 204 | 241 | 174 | 187 | 173 | 105 | 162 | 202 | 189 |
| QY36 |  | 204 | 241 | 174 | 187 | 173 | 105 | 162 | 202 | 189 |
| QY37 |  | 204 | 243 | 174 | 187 | 173 | 105 | 162 | 202 | 189 |
| QY38 |  | 204 | 243 | 174 | 187 | 173 | 105 | 162 | 202 | 189 |
| QY39 |  | 200 | 183 | 174 | 187 | 173 | 105 | 162 | 212 | 189 |
| QY40 |  | 130 | 199 | 147 | 187 | 187 | 103 | 162 | 212 | 185 |
| QY41 |  | 200 | 183 | 132 | 187 | 173 | 105 | 162 | 212 | 189 |
| QY42 |  | 200 | 183 | 174 | 187 | 173 | 105 | 162 | 212 | 189 |
| QY43 |  | 200 | 183 | 174 | 187 | 173 | 105 | 162 | 212 | 189 |
| QY44 |  | 204 | 241 | 174 | 187 | 173 | 105 | 162 | 202 | 189 |
| QY45 |  | 204 | 243 | 174 | 187 | 173 | 105 | 162 | 202 | 189 |
| QY46 |  | 200 | 183 | 174 | 187 | 173 | 105 | 162 | 212 | 189 |
| QY47 |  | 204 | 243 | 174 | 187 | 173 | 105 | 162 | 202 | 189 |
| QY48 |  | 128 | 243 | 174 | 187 | 173 | 105 | 162 | 202 | 189 |
| QY49 |  | 204 | 163 | 177 | 184 | 173 | 105 | 160 | 212 | 187 |
| MM1 | Maoming | 224 | 145 | 171 | 181 | 167 | 115 | 150 | 210 | 183 |
| MM2 |  | 166 | 145 | 171 | 181 | 167 | 105 | 160 | 212 | 187 |
| MM3 |  | 142 | 145 | 168 | 181 | 175 | 105 | 160 | 212 | 177 |
| MM4 |  | 142 | 145 | 177 | 187 | 173 | 105 | 160 | 212 | 169 |
| MM5 |  | 226 | 145 | 180 | 187 | 175 | 103 | 158 | 212 | 177 |
| MM6 |  | 160 | 145 | 171 | 187 | 167 | 103 | 160 | 212 | 169 |
| MM7 |  | 224 | 145 | 171 | 181 | 175 | 105 | 160 | 214 | 169 |
| MM8 |  | 162 | 145 | 171 | 187 | 175 | 105 | 160 | 212 | 169 |
| MM9 |  | 142 | 147 | 171 | 181 | 175 | 103 | 160 | 212 | 169 |
| MM10 |  | 162 | 147 | 171 | 187 | 167 | 103 | 160 | 212 | 169 |
| MM11 |  | 162 | 147 | 171 | 187 | 167 | 103 | 160 | 212 | 169 |
| MM12 |  | 162 | 147 | 171 | 187 | 167 | 103 | 160 | 212 | 169 |
| MM13 |  | 162 | 147 | 171 | 187 | 167 | 103 | 160 | 212 | 169 |
| YC1 | Yichun | 162 | 135 | 201 | 172 | 173 | 103 | 158 | 212 | 177 |
| YC2 |  | 162 | 135 | 201 | 187 | 173 | 101 | 158 | 212 | 177 |
| YC3 |  | 214 | 143 | 129 | 187 | 173 | 103 | 158 | 212 | 177 |
| YC4 |  | 144 | 143 | 129 | 187 | 171 | 103 | 158 | 212 | 177 |
| YC5 |  | 156 | 135 | 129 | 190 | 175 | 105 | 158 | 212 | 177 |
| YC6 |  | 156 | 145 | 183 | 184 | 173 | 103 | 162 | 202 | 177 |
| YC7 |  | 158 | 143 | 174 | 187 | 173 | 103 | 158 | 212 | 179 |
| YC8 |  | 150 | 145 | 177 | 187 | 173 | 103 | 158 | 212 | 177 |
| YC9 |  | 162 | 135 | 177 | 187 | 173 | 103 | 158 | 212 | 177 |
| YC10 |  | 158 | 143 | 174 | 172 | 173 | 101 | 158 | 212 | 177 |
| YC11 |  | 158 | 143 | 174 | 172 | 173 | 103 | 158 | 212 | 177 |
| YC12 |  | 124 | 143 | 168 | 187 | 173 | 105 | 158 | 212 | 177 |
| YC13 |  | 164 | 143 | 183 | 187 | 171 | 105 | 158 | 214 | 177 |
| YC14 |  | 128 | 143 | 168 | 187 | 173 | 105 | 158 | 212 | 177 |
| YC15 |  | 128 | 143 | 168 | 184 | 171 | 103 | 158 | 212 | 177 |
| YC16 |  | 128 | 143 | 168 | 187 | 173 | 105 | 158 | 212 | 177 |
| YC17 |  | 128 | 143 | 168 | 187 | 173 | 105 | 158 | 212 | 177 |
| YC18 |  | 128 | 143 | 168 | 187 | 173 | 105 | 158 | 212 | 177 |
| YC19 |  | 128 | 143 | 168 | 187 | 173 | 105 | 158 | 212 | 177 |
| YC20 |  | 128 | 143 | 168 | 187 | 173 | 105 | 158 | 212 | 177 |
| CQ1 | Chongqing | 168 | 145 | 171 | 172 | 173 | 105 | 158 | 212 | 177 |
| CQ2 |  | 168 | 143 | 168 | 172 | 173 | 105 | 158 | 212 | 177 |
| CQ3 |  | 158 | 145 | 156 | 172 | 173 | 105 | 160 | 212 | 177 |
| CQ4 |  | 156 | 145 | 156 | 172 | 173 | 105 | 160 | 212 | 177 |
| CQ5 |  | 168 | 145 | 171 | 172 | 173 | 105 | 158 | 212 | 177 |
| CQ6 |  | 156 | 145 | 156 | 172 | 173 | 105 | 160 | 212 | 177 |
| CQ7 |  | 156 | 145 | 156 | 172 | 173 | 105 | 160 | 212 | 177 |
| CQ8 |  | 156 | 145 | 156 | 172 | 173 | 105 | 160 | 212 | 177 |
| CQ9 |  | 156 | 145 | 156 | 172 | 173 | 105 | 160 | 212 | 177 |
| CQ10 |  | 156 | 145 | 156 | 172 | 173 | 105 | 160 | 212 | 177 |
| CQ11 |  | 170 | 143 | 168 | 172 | 173 | 105 | 158 | 212 | 177 |
| CQ12 |  | 156 | 145 | 156 | 172 | 173 | 105 | 160 | 212 | 177 |
| CQ13 |  | 168 | 173 | 168 | 175 | 175 | 105 | 154 | 212 | 177 |
| CQ14 |  | 156 | 145 | 156 | 172 | 173 | 105 | 160 | 212 | 177 |
| CQ15 |  | 156 | 145 | 156 | 172 | 173 | 105 | 160 | 212 | 177 |
| CQ16 |  | 156 | 145 | 156 | 172 | 173 | 105 | 160 | 212 | 177 |
| CQ17 |  | 156 | 145 | 156 | 172 | 173 | 105 | 160 | 212 | 177 |
| CQ18 |  | 156 | 145 | 156 | 172 | 173 | 105 | 160 | 212 | 177 |
| CQ19 |  | 156 | 145 | 156 | 172 | 173 | 105 | 160 | 212 | 177 |
| CQ20 |  | 156 | 145 | 156 | 172 | 173 | 105 | 160 | 212 | 177 |
| CQ21 |  | 156 | 145 | 156 | 172 | 173 | 105 | 160 | 212 | 177 |
| CQ22 |  | 156 | 145 | 156 | 172 | 173 | 105 | 160 | 212 | 177 |
| CQ23 |  | 156 | 145 | 156 | 172 | 173 | 105 | 160 | 212 | 177 |
| WH1 | Wuhan | 162 | 175 | 177 | 184 | 173 | 107 | 158 | 202 | 187 |
| WH2 |  | 202 | 143 | 168 | 187 | 173 | 105 | 158 | 214 | 177 |
| WH3 |  | 106 | 175 | 177 | 184 | 173 | 107 | 158 | 202 | 187 |
| WH4 |  | 162 | 145 | 174 | 187 | 173 | 105 | 158 | 214 | 185 |
| WH5 |  | 162 | 145 | 174 | 187 | 173 | 105 | 158 | 214 | 185 |
| WH6 |  | 162 | 145 | 174 | 187 | 173 | 105 | 158 | 214 | 185 |
| WH7 |  | 162 | 145 | 174 | 187 | 173 | 105 | 158 | 214 | 185 |
| WH8 |  | 162 | 145 | 174 | 187 | 173 | 105 | 158 | 214 | 185 |
| WH9 |  | 176 | 145 | 174 | 187 | 173 | 105 | 158 | 214 | 185 |
| WH10 |  | 162 | 145 | 174 | 187 | 173 | 105 | 158 | 214 | 185 |
| WH11 |  | 162 | 175 | 177 | 184 | 173 | 107 | 158 | 202 | 187 |
| WH12 |  | 150 | 133 | 129 | 178 | 171 | 97 | 150 | 202 | 175 |
| WH13 |  | 150 | 133 | 129 | 178 | 171 | 97 | 150 | 202 | 175 |
| WH14 |  | 162 | 145 | 174 | 187 | 173 | 105 | 158 | 214 | 185 |
| WH15 |  | 162 | 177 | 174 | 187 | 175 | 105 | 158 | 214 | 185 |
| WH16 |  | 162 | 145 | 174 | 187 | 173 | 105 | 158 | 214 | 185 |
| WH17 |  | 162 | 145 | 174 | 187 | 173 | 105 | 158 | 214 | 185 |
| WH18 |  | 162 | 145 | 174 | 187 | 173 | 105 | 158 | 214 | 185 |
| WH19 |  | 162 | 145 | 174 | 187 | 173 | 105 | 158 | 214 | 185 |
| WH20 |  | 162 | 145 | 174 | 187 | 173 | 105 | 158 | 214 | 185 |
| WH21 |  | 162 | 145 | 174 | 187 | 173 | 105 | 158 | 214 | 185 |
| WH22 |  | 162 | 145 | 174 | 187 | 173 | 105 | 158 | 214 | 185 |
| WH23 |  | 162 | 145 | 174 | 187 | 173 | 105 | 158 | 214 | 185 |
| WH24 |  | 162 | 145 | 174 | 187 | 173 | 105 | 158 | 214 | 185 |
| WH25 |  | 162 | 145 | 174 | 187 | 173 | 105 | 158 | 214 | 185 |
| FX1 | Fengxiang | 144 | 145 | 183 | 184 | 179 | 105 | 160 | 212 | 177 |
| FX2 |  | 144 | 145 | 129 | 184 | 179 | 105 | 160 | 212 | 177 |
| FX3 |  | 150 | 145 | 174 | 184 | 179 | 105 | 160 | 212 | 177 |
| FX4 |  | 144 | 143 | 174 | 187 | 173 | 103 | 160 | 212 | 187 |
| FX5 |  | 144 | 143 | 174 | 187 | 173 | 103 | 160 | 212 | 187 |
| FX6 |  | 144 | 145 | 162 | 187 | 179 | 105 | 160 | 212 | 177 |
| FX7 |  | 144 | 145 | 162 | 184 | 179 | 105 | 160 | 212 | 177 |
| FX8 |  | 144 | 143 | 174 | 187 | 173 | 103 | 160 | 212 | 187 |
| FX9 |  | 144 | 143 | 174 | 187 | 173 | 103 | 160 | 212 | 187 |
| FX10 |  | 144 | 143 | 174 | 187 | 173 | 103 | 160 | 212 | 187 |
| FX11 |  | 144 | 143 | 174 | 187 | 173 | 103 | 160 | 212 | 187 |
| FX12 |  | 144 | 143 | 174 | 187 | 173 | 103 | 160 | 212 | 187 |
| WC1 | Wucheng | 158 | 165 | 177 | 184 | 173 | 105 | 160 | 212 | 187 |
| WC2 |  | 160 | 161 | 129 | 184 | 171 | 105 | 160 | 212 | 179 |
| WC3 |  | 128 | 133 | 168 | 181 | 167 | 107 | 150 | 210 | 211 |
| WC4 |  | 128 | 139 | 129 | 178 | 165 | 105 | 150 | 210 | 187 |
| WC5 |  | 128 | 133 | 129 | 178 | 173 | 105 | 150 | 212 | 179 |
| WC6 |  | 128 | 133 | 129 | 178 | 167 | 105 | 150 | 210 | 177 |
| WC7 |  | 202 | 145 | 129 | 184 | 173 | 105 | 158 | 212 | 177 |
| WC8 |  | 202 | 143 | 168 | 184 | 173 | 105 | 158 | 212 | 175 |
| WC9 |  | 160 | 165 | 129 | 184 | 173 | 105 | 160 | 212 | 179 |
| WC10 |  | 128 | 133 | 168 | 178 | 167 | 105 | 150 | 210 | 187 |
| WC11 |  | 162 | 163 | 129 | 184 | 173 | 105 | 154 | 202 | 179 |
| WC12 |  | 128 | 133 | 129 | 181 | 171 | 109 | 150 | 210 | 179 |
| WC13 |  | 128 | 133 | 129 | 187 | 173 | 105 | 160 | 210 | 179 |
| WC14 |  | 128 | 133 | 177 | 178 | 167 | 105 | 150 | 210 | 187 |
| WC15 |  | 158 | 163 | 204 | 184 | 173 | 105 | 160 | 212 | 187 |
| WC16 |  | 158 | 163 | 177 | 184 | 173 | 105 | 160 | 212 | 187 |
| WC17 |  | 200 | 143 | 129 | 187 | 173 | 103 | 160 | 212 | 187 |
| WC18 |  | 156 | 163 | 129 | 184 | 173 | 105 | 160 | 212 | 175 |
| WC19 |  | 202 | 145 | 174 | 184 | 173 | 105 | 158 | 212 | 181 |
| WC20 |  | 128 | 133 | 129 | 172 | 167 | 115 | 150 | 210 | 183 |
| WC21 |  | 158 | 165 | 180 | 184 | 173 | 105 | 160 | 212 | 187 |
| WC22 |  | 128 | 139 | 129 | 178 | 163 | 105 | 150 | 210 | 205 |
| WC23 |  | 128 | 133 | 129 | 178 | 167 | 105 | 150 | 210 | 179 |
| WC24 |  | 128 | 133 | 129 | 178 | 167 | 107 | 150 | 210 | 179 |
| WC25 |  | 128 | 133 | 129 | 178 | 167 | 105 | 150 | 210 | 179 |
| WC26 |  | 128 | 139 | 129 | 178 | 163 | 105 | 150 | 210 | 205 |
| WC27 |  | 158 | 133 | 129 | 178 | 167 | 107 | 150 | 210 | 179 |
| WC28 |  | 204 | 165 | 183 | 184 | 173 | 105 | 160 | 212 | 187 |
| WC29 |  | 128 | 139 | 129 | 178 | 163 | 105 | 150 | 210 | 205 |
| WC30 |  | 128 | 133 | 129 | 178 | 167 | 107 | 150 | 210 | 179 |
| WC31 |  | 128 | 133 | 129 | 178 | 167 | 105 | 150 | 210 | 179 |
| WC32 |  | 204 | 133 | 129 | 178 | 165 | 105 | 150 | 210 | 219 |
| WC33 |  | 204 | 133 | 129 | 178 | 167 | 107 | 150 | 210 | 179 |
| WC34 |  | 128 | 133 | 129 | 187 | 173 | 105 | 162 | 202 | 189 |
| WC35 |  | 128 | 133 | 129 | 172 | 167 | 115 | 150 | 210 | 183 |
| WC36 |  | 160 | 133 | 129 | 172 | 167 | 109 | 150 | 210 | 179 |
| WC37 |  | 128 | 179 | 174 | 187 | 173 | 105 | 162 | 202 | 189 |
| WC38 |  | 130 | 133 | 129 | 178 | 167 | 107 | 150 | 210 | 179 |
| WC39 |  | 128 | 139 | 129 | 178 | 163 | 105 | 150 | 210 | 205 |
| WC40 |  | 128 | 139 | 129 | 178 | 163 | 105 | 150 | 210 | 205 |
| WC41 |  | 128 | 139 | 129 | 178 | 163 | 105 | 150 | 210 | 205 |
| WC42 |  | 128 | 139 | 129 | 178 | 163 | 105 | 150 | 210 | 205 |
| WC43 |  | 128 | 139 | 129 | 178 | 163 | 105 | 150 | 210 | 205 |
| LY1 | Laiyang | 128 | 149 | 129 | 178 | 163 | 105 | 150 | 210 | 221 |
| LY2 |  | 128 | 139 | 129 | 178 | 163 | 105 | 150 | 210 | 203 |
| LY3 |  | 128 | 149 | 129 | 178 | 163 | 105 | 150 | 210 | 221 |
| LY4 |  | 128 | 139 | 129 | 178 | 165 | 105 | 150 | 210 | 213 |
| LY5 |  | 128 | 139 | 129 | 178 | 165 | 105 | 150 | 210 | 213 |
| LY6 |  | 128 | 139 | 129 | 178 | 165 | 105 | 150 | 210 | 213 |
| LY7 |  | 128 | 139 | 129 | 178 | 165 | 105 | 150 | 210 | 213 |
| LY8 |  | 128 | 139 | 129 | 178 | 165 | 105 | 150 | 210 | 213 |
| LY9 |  | 128 | 139 | 129 | 178 | 165 | 105 | 150 | 210 | 213 |
| LY10 |  | 128 | 139 | 129 | 178 | 165 | 105 | 150 | 210 | 213 |
| TJ1 | Tianjin | 128 | 149 | 177 | 178 | 163 | 105 | 150 | 210 | 175 |
| TJ2 |  | 128 | 151 | 129 | 178 | 167 | 111 | 150 | 210 | 207 |
| TJ3 |  | 128 | 151 | 177 | 178 | 165 | 105 | 150 | 210 | 203 |
| TJ4 |  | 128 | 139 | 168 | 178 | 163 | 105 | 150 | 210 | 179 |
| TJ5 |  | 128 | 133 | 129 | 178 | 163 | 111 | 150 | 210 | 205 |
| TJ6 |  | 128 | 151 | 129 | 178 | 165 | 105 | 150 | 210 | 211 |
| TJ7 |  | 128 | 141 | 177 | 178 | 165 | 105 | 152 | 210 | 179 |
| TJ8 |  | 128 | 133 | 174 | 178 | 163 | 111 | 150 | 210 | 169 |
| TJ9 |  | 128 | 133 | 174 | 178 | 163 | 111 | 150 | 210 | 169 |
| TJ10 |  | 128 | 133 | 174 | 178 | 163 | 111 | 150 | 210 | 169 |
| TJ11 |  | 128 | 133 | 174 | 178 | 163 | 111 | 150 | 210 | 169 |
| LF1 | Langfang | 150 | 133 | 129 | 178 | 169 | 97 | 150 | 202 | 175 |
| LF2 |  | 150 | 133 | 129 | 178 | 171 | 95 | 150 | 202 | 175 |
| LF3 |  | 128 | 133 | 129 | 187 | 165 | 105 | 150 | 210 | 179 |
| LF4 |  | 150 | 133 | 129 | 178 | 167 | 97 | 150 | 202 | 175 |
| LF5 |  | 150 | 133 | 129 | 178 | 183 | 95 | 150 | 202 | 175 |
| LF6 |  | 150 | 133 | 129 | 178 | 183 | 95 | 150 | 202 | 175 |
| LF7 |  | 150 | 133 | 129 | 178 | 171 | 95 | 150 | 204 | 175 |
| LF8 |  | 128 | 133 | 129 | 175 | 161 | 105 | 150 | 210 | 179 |
| LF9 |  | 124 | 133 | 129 | 178 | 173 | 97 | 150 | 202 | 175 |
| LF10 |  | 124 | 133 | 171 | 178 | 173 | 97 | 150 | 202 | 175 |
| LF11 |  | 124 | 133 | 171 | 178 | 173 | 95 | 150 | 202 | 175 |
| LF12 |  | 128 | 133 | 129 | 181 | 163 | 105 | 150 | 210 | 179 |
| LF13 |  | 128 | 133 | 129 | 178 | 165 | 167 | 156 | 216 | 175 |
| LF14 |  | 124 | 133 | 129 | 178 | 165 | 97 | 150 | 202 | 175 |
| LF15 |  | 150 | 133 | 129 | 178 | 165 | 97 | 150 | 202 | 175 |
| LF16 |  | 150 | 133 | 129 | 178 | 183 | 95 | 150 | 202 | 175 |
| LF17 |  | 150 | 133 | 129 | 178 | 183 | 95 | 150 | 202 | 175 |
| LF18 |  | 150 | 133 | 129 | 178 | 183 | 95 | 150 | 202 | 175 |
| LF19 |  | 150 | 133 | 129 | 178 | 183 | 95 | 150 | 202 | 175 |
| LF20 |  | 150 | 133 | 129 | 178 | 183 | 95 | 150 | 202 | 175 |
| BJ1 | Beijing | 150 | 133 | 183 | 178 | 183 | 95 | 150 | 202 | 175 |
| BJ2 |  | 150 | 133 | 174 | 178 | 183 | 97 | 150 | 202 | 175 |
| BJ3 |  | 150 | 133 | 201 | 178 | 183 | 95 | 150 | 202 | 175 |
| BJ4 |  | 124 | 133 | 129 | 178 | 173 | 97 | 150 | 202 | 175 |
| BJ5 |  | 150 | 133 | 129 | 178 | 183 | 97 | 150 | 202 | 175 |
| BJ6 |  | 150 | 133 | 129 | 178 | 183 | 95 | 150 | 202 | 175 |
| BJ7 |  | 150 | 133 | 168 | 178 | 183 | 97 | 150 | 202 | 175 |
| BJ8 |  | 150 | 133 | 129 | 178 | 183 | 97 | 150 | 208 | 175 |
| BJ9 |  | 150 | 133 | 168 | 178 | 183 | 95 | 150 | 202 | 203 |
| BJ10 |  | 150 | 133 | 129 | 178 | 183 | 97 | 150 | 202 | 175 |
| BJ11 |  | 124 | 133 | 129 | 178 | 167 | 97 | 150 | 202 | 211 |
| BJ12 |  | 128 | 139 | 102 | 178 | 163 | 105 | 150 | 210 | 175 |
| BJ13 |  | 124 | 133 | 204 | 178 | 167 | 97 | 150 | 202 | 175 |
| BJ14 |  | 128 | 139 | 177 | 178 | 163 | 105 | 150 | 210 | 187 |
| BJ15 |  | 150 | 133 | 129 | 178 | 183 | 97 | 150 | 202 | 175 |
| BJ16 |  | 150 | 133 | 129 | 178 | 183 | 97 | 150 | 202 | 175 |
| BJ17 |  | 150 | 133 | 129 | 178 | 183 | 97 | 150 | 202 | 175 |
| BJ18 |  | 150 | 133 | 129 | 178 | 183 | 97 | 150 | 202 | 175 |
| BJ19 |  | 150 | 133 | 129 | 178 | 183 | 97 | 150 | 202 | 175 |
| XC1 | Xingcheng | 128 | 139 | 129 | 178 | 165 | 105 | 150 | 210 | 205 |
| XC2 |  | 128 | 139 | 129 | 184 | 163 | 105 | 150 | 210 | 205 |
| XC3 |  | 128 | 139 | 129 | 178 | 165 | 105 | 152 | 210 | 211 |
| XC4 |  | 128 | 133 | 129 | 175 | 163 | 105 | 150 | 210 | 179 |
| XC5 |  | 128 | 149 | 129 | 178 | 161 | 105 | 150 | 210 | 215 |
| XC6 |  | 128 | 139 | 129 | 187 | 163 | 105 | 150 | 210 | 205 |
| XC7 |  | 128 | 139 | 129 | 178 | 165 | 105 | 152 | 210 | 211 |
| XC8 |  | 128 | 139 | 129 | 178 | 165 | 105 | 152 | 210 | 211 |
| XC9 |  | 128 | 133 | 129 | 178 | 161 | 101 | 150 | 210 | 179 |
| XC10 |  | 128 | 149 | 129 | 184 | 159 | 105 | 164 | 210 | 215 |
| XC11 |  | 128 | 139 | 129 | 178 | 165 | 105 | 152 | 210 | 211 |
| XC12 |  | 128 | 139 | 129 | 178 | 165 | 105 | 152 | 210 | 211 |
| XC13 |  | 128 | 139 | 129 | 178 | 165 | 105 | 152 | 210 | 211 |
| XC14 |  | 128 | 139 | 129 | 178 | 165 | 105 | 152 | 210 | 211 |
| XC15 |  | 128 | 139 | 129 | 178 | 165 | 105 | 152 | 210 | 211 |
| XC16 |  | 128 | 139 | 129 | 178 | 165 | 105 | 152 | 210 | 211 |
| CC1 | Changchun | 150 | 133 | 174 | 187 | 163 | 105 | 150 | 202 | 175 |
| CC2 |  | 128 | 133 | 138 | 187 | 173 | 85 | 150 | 210 | 189 |
| CC3 |  | 160 | 147 | 177 | 184 | 173 | 105 | 160 | 212 | 187 |
| CC4 |  | 128 | 141 | 171 | 190 | 163 | 85 | 150 | 210 | 175 |
| CC5 |  | 128 | 141 | 129 | 178 | 161 | 105 | 150 | 210 | 203 |
